# Guidelines for Orthodontic Management of Individuals With Mental Illness Using Psychiatric Medication: A Systematic Review

**DOI:** 10.7759/cureus.40604

**Published:** 2023-06-18

**Authors:** AbdulMajeed A AlMogbel, Abdulatif Aldahami, Shahad Al Numair, Khluod M Alkhowailed, Ali Al Numair

**Affiliations:** 1 Orthodontics and Pediatric Dentistry, Qassim University, Buraydah, SAU; 2 Dental Surgery, Qassim University, Buraydah, SAU; 3 Pharmacy, Qassim University, Buraydah, SAU

**Keywords:** evidence-based dentistry, systematic review, special health care needs, orthodontic guidelines, mental illness, intravenous sedation, behavior modification

## Abstract

The majority of mentally challenged individuals anticipate treatment with inflated levels of concern more than conventional orthodontic patients. However, there are no systematic reviews on behavioral modification techniques and orthodontic therapy for people with mental illness. Therefore, the goal of the review was to highlight the orthodontic concerns for people with mental disabilities with the intent to address the problems that emerge when providing orthodontic care. The Preferred Reporting Items for Systematic Reviews and Meta-Analyses (PRISMA) guidelines and population, exposure, and outcome (PEO) criteria were followed in conducting the review. An electronic search was performed in the PubMed, Scopus, EMBASE, Cochrane Oral Health Group, and Dentistry and Oral Science Source databases searched through EBSCO and Google Scholar for potentially relevant publications in the English language from January 2002 to December 2022. Studies reporting behavioral modification strategies and/or physical constraints used during orthodontic treatment of mentally challenged patients were included in the review. The quality of the included studies was evaluated using the Joanna Briggs Institute (JBI) critical appraisal checklist for case reports and research reviews and synthesis. The initial electronic and manual search yielded 233 articles. After eliminating duplicates and reviewing the title/abstracts, 75 articles were selected for independent full-text review. Based on the eligibility criteria, 12 studies were finally chosen for qualitative synthesis. Four of these studies were case reports, while eight were comprehensive reviews. The JBI critical assessment criteria for case reports revealed that two studies had moderate-quality evidence, one case report with high-quality evidence and the other with low-quality evidence. The quality of the selected comprehensive literature reviews assessed using JBI critical assessment for reviews and research syntheses was judged to have poor-quality evidence. A thorough literature search on the topic did not reveal a single systematic review, and all of the reviews that were chosen were exhaustive. Parental cooperation and patient motivation are crucial components of a successful treatment regimen. A better prognosis is determined by the choice of appropriate orthodontic mechanotherapy along with the utilization of an array of behavior modification modalities and the availability of a team with expertise.

## Introduction and background

Adolescents and children with special healthcare needs (SHCN) have a higher prevalence of malocclusion than the general population. Any physical, developmental, mental, sensory, behavioral, cognitive, or emotional dysfunction or restricting condition that necessitates medical management, healthcare assistance, and/or the utilization of specialized amenities is classified as an individual with SHCN. The number of children and adolescents with SHCN has been growing steadily through the decades as a result of population growth, longer life expectancy, and improved access to healthcare, as well as more precise and perceptual methods of early identification and diagnosis [1].

When people with disabilities indicate an attempt to seek out health services and are successful in accomplishing so, they then decide to continue receiving treatment from experts who are sympathetic, caring, and accountable. Accessibility to care, however, is thought to be the primary obstacle to the commencement of a health service, as the condition of the patient should be comprehended and their therapeutic trajectory in the continuum of services ought to be tracked as their most fundamental and SHCN [2]. One out of every five children in the United States of America (USA) was estimated to be individuals with SHCN [3]. The pilot research conducted in India in 2002 found that more than 25% of patients undergoing orthodontic treatment had an ailment that might have affected their treatment. An orthodontist in practice should be well-equipped to handle the difficulties associated with identifying and addressing the SHCN population [4].

The American Association of Mental Deficiency (AAMD) referred the mental retardation as those individuals below the average level of general intellectual functioning emanating during the developmental phase and associated with an adaptive behavioral disability. According to their level of intelligence, the AAMD divides retardation into four distinct groups: mild, moderate, severe, and profound. A person is deemed to have mild mental retardation if their intelligence quotient (IQ) score is between 50 and 70; moderate retardation if it is between 35 and 50; severe retardation if it is between 20 and 35; and profound retardation if it is below 20. Patients with Down syndrome are typically referred to as profoundly retarded because their IQ ranges from 25 to 50 [5]. Autism and pervasive developmental disorder not otherwise defined in the Diagnostic and Statistical Manual of Mental Disorders IV are included in the new DSM-5, and those conditions are referred to as autism spectrum disorder (ASD) [6].

Using non-pharmacologic behavior management techniques like the tell-show-do method, the majority of individuals with SHCN can be successfully managed. The treatment of disabled individuals with profound dental problems frequently requires the use of pharmacological behavior management approaches, such as nitrous oxide/oxygen sedation, or general anesthesia (GA), in achieving higher-quality healthcare [7].

The majority of challenged children anticipate treatment with inflated levels of concern more than conventional orthodontic patients. Therefore, to acquire their trust, these patients must be treated with empathy and understanding [8]. However, there are no systematic reviews on behavioral modification techniques and orthodontic therapy for people with mental illness. Therefore, the goal of the review was to highlight the orthodontic concerns for people with mental disabilities with the intent to address the problems that emerge when providing orthodontic care.

## Review

Material and method

The Preferred Reporting Items for Systematic Reviews and Meta-Analyses (PRISMA) guidelines were followed in conducting the review [9]. Based on the population, exposure, and outcome (PEO) criteria, a structured question was laid out. Population: People with mental illness seeking orthodontic treatment. Exposure: Use of behavioral modification approaches or physical restraints. Results: Orthodontic care for those with mental illness. Focused question: Do specific orthodontic treatment guidelines facilitate patients with mental illness to attain their intended clinical outcomes?

Search Strategy

An electronic search was performed in the PubMed, Scopus, EMBASE, Cochrane Oral Health Group, and Dentistry and Oral Science Source databases searched through EBSCO for potentially relevant publications in the English language from January 2002 to December 2022. The following Medical Subject Headings (MeSH) phrases were employed: ("Special Healthcare Needs" OR "Disabled Patients" OR "Mental Retardation" OR "Mentally Challenged" OR "Cerebral Palsy" OR "Autism" OR "Down Syndrome" OR "Differentially Abled Individuals" AND ("Orthodontic Treatment" OR "Orthodontic Guidelines' OR "Malocclusion" OR "Therapeutic Planning" OR "Orthodontic Considerations" OR "Orthodontic Recommendations"). A manual search of journals was combined with an electronic search to identify appropriate articles. Google Scholar was utilized to retrieve any articles not found in the above-mentioned databases while searching for grey literature.

Selection Criteria of Eligible Studies

Studies have to meet the following inclusion criteria to be included in the systematic review: Studies reporting behavioral modification strategies and/or physical constraints used during orthodontic treatment on mentally challenged patients in case reports, case series, and randomized or non-randomized prospective clinical trials were included in the review. The search was restricted to English-language publications that had no age or gender limitations. Due to the limited amount of studies that have been done in this area of interest, the review covered all possible research design types. Editorials, professional opinions, and technical reports that were published before 2001 were, however, excluded.

Study Selection

Each title and abstract were evaluated initially independently by two reviewers. The studies that cleared the initial screening underwent full-text analysis after meeting the inclusion criteria. Additionally, other pertinent published papers were searched in the reference lists of the chosen studies. A third reviewer was sought out to confirm the eligibility for the review if there were any inconsistencies between the two reviewers.

Quality Assessment

Using a yes/no, unclear, or not applicable questionnaire format, the quality of the included case reports was evaluated using the Joanna Briggs Institute (JBI) critical appraisal checklist for case reports, which covers eight important study-related parameters [10]. Case reports that received a score of four or less were considered to have low-quality evidence, while those that received a score of seven to eight were considered to have evidence of high quality.

The JBI critical evaluation checklist for research syntheses, which examines 11 essential features of the review, was used to evaluate the quality of the chosen comprehensive reviews [11]. A review that received a maximum score of 11 was considered to be competent. Studies with 7 to 10 points were deemed to exhibit moderate quality and those with 0 to 6 points as having poor-quality evidence [6]. Two reviewers made decisions about the suitability and eligibility of the study, and any discrepancies were worked out through discussion.

Data Extraction

For further qualitative analysis, two independent reviewers gathered data from the studies that were taken into account and related to the important predetermined outcomes. The names of the authors, the year of publication, the study design, sample size, demographic characteristics (age and gender, if available), the type of disability, the adjunctive treatment method used, the clinical outcome, the suggested orthodontic treatment guidelines, and the conclusion of the chosen studies were taken from the studies that were included. Conflicts in decisions were resolved as was previously mentioned through discussion. The respective authors of the research were contacted if more information regarding the study methods, any uncertainties, or any specific data were required.

Result

Study Selection

**Figure 1:**
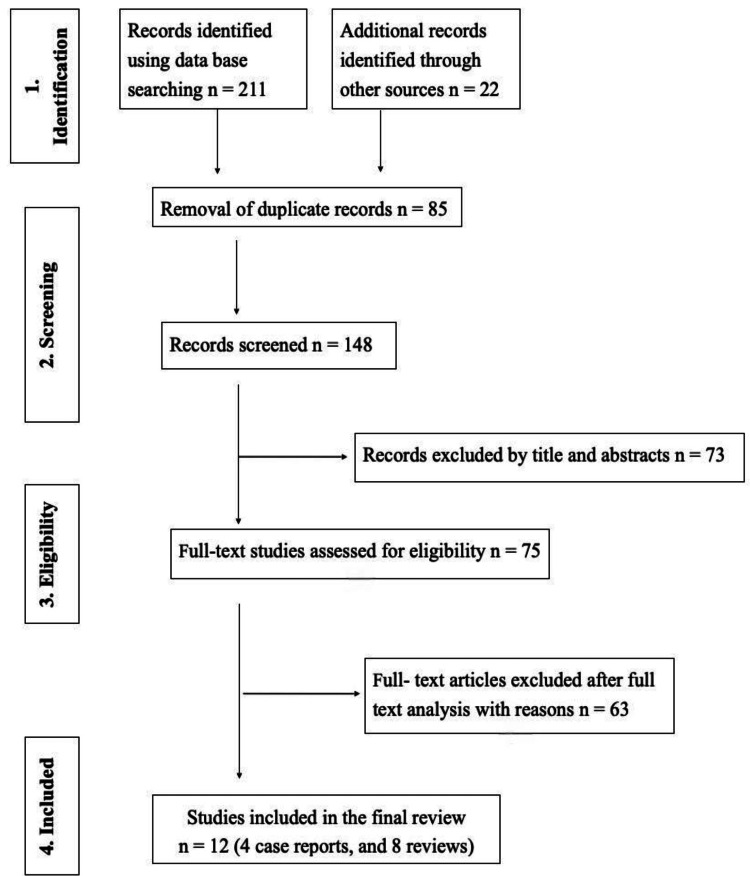
PRISMA flowchart of the included studies (adapted from the 2009 PRISMA flow diagram)

The initial electronic search yielded 211 articles according to the PRISMA guidelines (Figure 1). An additional 22 articles were identified with manual search with a collection of a total of 233 articles for review. After eliminating duplicates, 148 papers were available for screening. The abstracts of 73 studies were excluded after reviewing the title and abstracts, and 75 articles were selected for independent full-text review by two investigators. After screening the remaining studies based on the eligibility criteria, 63 articles were excluded, and 12 studies were finally chosen for qualitative synthesis. Four of these studies [12-15] were case reports, while eight were comprehensive reviews [16-23]. For title/abstract screening and full-text evaluation, Kappa scores for inter-examiner reliability were 0.84 and 0.90, respectively, for screening of abstract or title and full-text reading. A third reviewer was consulted to resolve any discrepancies between the investigators. Meta-analysis was not feasible due to the heterogeneity of the studies.

Characteristics of Included Studies

The current systematic review consisted of four case reports and eight comprehensive literature reviews. Table 1 illustrates the key traits of the case reports that have been reviewed. The overview of findings from the chosen comprehensive reviews on orthodontic recommendations for individuals with mental illness is shown in Table 2.

**Table 1:** Summary characteristics of case reports included in the review RPE: rapid palatal expander; GA: general anesthesia; SHCN: special healthcare needs

Author-year	Country	Age (years)	Gender (n)	Study design	Diagnosis	Adjunctive modalities	Orthodontic treatment	Clinical outcome and recommended guidelines
Sabuncuoglu, et al., 2014 [12]	Turkey	10	Male	Case report	Cerebral Palsy	Desensitization	Twin blocks with a headgear followed by fixed appliance	The success of the orthodontic treatment depends on the patient's and parent's compliance
Rada et al., 2014 [13]	Illinois, USA	12 years and 8 years	Female 1, male 1	Case reports	One each with cerebral palsy and autism	GA	Cephalometric X-ray and fixed palatal expander	Compliance and modification of the treatment plan as required will play a beneficial role in a successful clinical outcome for individuals with an intellectual and developmental disability
Hobson et al., 2005 [14]	London, UK	11 to 13	Male 1, female 4	Case series	One case each of Rubinstein-Taybi syndrome, Prader-Willi syndrome, and Cri-du-chat syndrome, two cases of cerebral palsy	GA	Extraction followed by bonding of the fixed appliance, bonding of retainers	Children with SHCN require a pre-assessment appraisal period. It is important to keep parents and guardians informed about treatment rationale and their contribution to accomplishing the intended objectives. The use of behavior management approaches will make it possible to gauge an individual's level of tolerance and endurance to exposure to diagnostic radiography. Although GA might be advantageous for physical restraints, it should not be used unless alternative management techniques have been explored
Chaushu et al., 2002 [15]	Jerusalem, Israel	13 ± 3.8 (9-23)	Female 7, male 3	Case reports	Five cases of mental retardation, two with cerebral palsy, one each with Down syndrome, and severe dental phobia	Deep intravenous sedation with propofol and midazolam	Bonding of brackets, debonding of RPE, cementation of molar bands, radiographs, and impression-making	Individuals with SHCN can receive orthodontic treatment on a more flexible schedule with less morbidity when intravenous sedation with propofol is used

**Table 2:** Summary of the conclusion of the selected comprehensive reviews published on orthodontic guidelines for mentally disabled individuals ASD: autism spectrum disorder; ADHD: attention-deficit hyperactivity disorder; GA: general anesthesia; SHCN: special healthcare needs

Author-year	Conclusion of the review
Dasgupta et al., 2021 [16]	The orthodontist should pay particular attention to hand gestures and verbal demands because people with mental illness frequently struggle with both verbal and nonverbal interactions. Techniques for changing behavior include progression, backward chaining, hypnosis, kinesthesia, and audio analgesia. It is also possible to use GA or conscious/IV sedation as the pharmacological technique of management. It is also possible to employ protective stabilization, such as Velcro straps, a papoose board, or a head stabilizer.
Erwansyah et al., 2020 [17]	To improve treatment outcomes, effective interaction should be developed between the orthodontist, parents, and patients. In cases of severe mental impairment, GA is required for the initial placement of fixed appliances, bonded, or cemented bite planes.
Alqahtani et al., 2019 [18]	Tell-show-do, desensitization, positive reinforcement, and protective stabilization strategies can all be used during certain procedures. Sedation or GA may be used in some circumstances. For patients with ASD and ADHD, removable orthodontic equipment should be small and wire-reinforced. Patient cooperation would be improved by brief appointments with regular interruptions.
Soto et al., 2017 [19]	For the treatment to be deemed successful, interactions between the healthcare professional and the parents will be essential. Based on the skeletal and dental dysplasia, orthodontic therapy is recommended together with behavior management procedures like the Tell-show-do procedure and/or distraction osteogenesis.
Dash et al., 2016 [20]	Based on each patient's potential for cooperation, customized treatment plans should be established. For the treatment of severe malocclusion in mentally challenged individuals, orthognathic surgeries may be a viable choice.
Arora et al., 2013 [21]	By setting realistic goals, using behavior modification techniques, delivering treatment in modules to streamline the course of treatment, and using deep IV sedation or GA, along with active retention appliances like removable total acrylic high-pull integral headgear for post-treatment retention. Conventional orthodontic guidelines can be modified to meet the individualized treatment objectives of patients with SHCN.
Goenharto et al., 2012 [22]	With clear explanations and effective communication, orthodontic therapy could be provided to selective Down syndrome patients. To further achieve the goals of the therapy, an experienced orthodontist and chairside assistant with specialized facilities like sedation equipment for conscious sedation, intravenous sedation, or GA are needed.
Becker et al., 2004 [23]	To satisfy the needs of the particular treatment each patient requires, the least complicated and the most prudent behavior modification strategies should be applied. When those strategies fail to achieve therapeutic accessibility, modalities like conscious sedation or GA should be utilized as an adjuvant.

Sabuncuoglu et al. [12] used a desensitization procedure with a six-year follow-up to demonstrate the use of twin blocks with headgear in a 10-year-old boy with profound class II skeletal dysplasia. A subsequent fixed appliance was found to be acceptable by the patient. In the treatment of a 12-year-old girl with cerebral palsy and an 8-year-old boy with autism, Rada et al. [13] reportedly considered orthodontic guidelines for SHCN into account. GA was used to carry out orthodontic procedures in both cases. It was further said that a fellowship-based training program will help to achieve outstanding standards of orthodontic care. According to Hobson et al. [14], behavior management approaches may be necessary for patients to accept and be able to handle therapeutic procedures, as well as to be exposed to diagnostic radiography. Even though a child's behavior can alter as they grow older, the type of management strategies used will also depend on the operator's comfort and confidence. Before considering orthodontic treatment for this particular group of individuals, parents had to be demonstrated regarding the measures of maintaining the oral hygiene of their children. When providing orthodontic therapy to individuals with mental retardation, Chaushu et al. [15] emphasized the use of intravenous sedation as an alternative to GA.

Dasgupta et al. [16] conducted an in-depth investigation with a range of treatments including psychosocial, pharmacological, and physical constraints to provide the best orthodontic care for people with mental retardation depending on their unique needs. Alqahtani et al. [18] evaluated the recommendations and changes in the treatment plan to enable these patients to benefit from therapeutic services and discussed the orthodontic management of attention-deficit hyperactivity disorder (ADHD) and ASD. It takes a great deal of work to provide orthodontic care for young children with mental illness, and it must be implemented in several sessions. It is advised to consult with the child's parents or guardians to determine their preferred educational strategies and apply them in practice. To acquire the patient's confidence and compliance, techniques such as giving brief, clear instructions, voice control, the tell-show-do method, behavior modification, and positive reinforcement can also be used.

Soto et al. [19] suggested that the orthodontist be aware of potential avenues along with alterations in standard orthodontic and orthopedic interventions to address cases with SHCN and to enhance these patients' utilization of healthcare services. Interaction between the experts and the parents will be critical for treatment to be considered successful even if not each course of treatment goal is met or even if the patient is not compliant. Erwansyah et al. [17] and Dash et al. [20] discussed essential variables that must be weighed when planning and providing orthodontic care to children with mental retardation, Down syndrome, and cerebral palsy. Specific goal-driven orthodontic care will enhance the patient's functional and aesthetically appealing outcome. Furthermore, it was suggested that scheduling a brief appointment with stabilizing the head and refraining from an entirely reclined position in the dental chair during treatment will foster the treatment objective.

Goenharto et al. [22] evaluated the difficulties that confronted patients with Down syndrome and suggested different behavioral management methods for people with SHCN. The three fundamental concepts of the orthodontic management of mentally disabled children, according to Arora et al. [21] and Becker et al., [23] are to increase the patient's degree of confidence in the clinical setting, determine the patient's and parental compliance with personal care at home, and investigate the anticipated extent of cooperation that will eventually be inevitable. Furthermore, it was mentioned that the panoramic radiograph has been demonstrated to be beneficial for orthodontic assessment because some patients might be apprehensive about having a periapical radiograph taken. A computed tomography scan may be carried out while the patient is being sedated in some circumstances.

Quality Assessment of the Reviewed Studies

The quality of the chosen case reports was evaluated using the JBI critical assessment criteria for case reports. While the study by Hobson et al. [14] and Chaushu et al. [15] received a score of six and five, respectively, indicating moderate quality, the study by Sabuncuoglu et al. [12] received a score of seven, demonstrating good-quality evidence. The case report by Rada et al. [13] received a score of four out of eight, suggesting low-quality evidence (Table 3).

**Table 3:** JBI critical appraisal checklist for case reports Sabuncuoglu et al., 2014 [12], Rada et al., 2014 [13], Hobson et al., 2005 [14], Chaushu et al., 2002 [15]

	Sabuncuoglu et al., 2014 [12]	Rada et al. 2014 [13]	Hobson et al. 2005 [14]	Chaushu et al. 2002 [15]
Were the patient’s demographic characteristics clearly described?	Yes	Yes	Yes	Yes
Was the patient’s history clearly described and presented as a timeline?	Yes	No	Yes	Unclear
Was the current clinical condition of the patient on presentation clearly described?	Yes	No	Yes	Yes
Were diagnostic tests or assessment methods and the results clearly described?	Yes	No	No	No
Was the intervention(s) or treatment procedure(s) clearly described?	Yes	Yes	Yes	Yes
Was the post-intervention clinical condition clearly described?	Yes	Yes	Yes	Yes
Were adverse events (harms) or unanticipated events identified and described?	No	No	No	No
Does the case report provide takeaway lessons?	Yes	Yes	Yes	Yes
Total	7	4	6	5

The quality of the selected comprehensive reviews of the literature was assessed using the JBI critical assessment for reviews and research syntheses. The investigations carried out by Soto et al. [19] and Alqahtani et al. [18] received scores of five and three, respectively. The remaining evaluated reviews were assigned a rating of two. All the included literature studies in the current systematic review were judged to have poor-quality evidence. A thorough search of the literature on the topic did not reveal a single systematic review, and all of the reviews that were chosen were exhaustive. Additionally, neither of the reviews explicitly indicated either the research topic or the criteria to be considered for the process of study selection (Table 4).

**Table 4:** JBI critical appraisal quality assessment checklist of the retrieved reviews and research syntheses Dasgupta et al., 2021 [16], Erwansyah et al., 2020 [17], Alqahtani et al., 2019 [18], Soto et al., 2017 [19], Dash et al., 2016 [20], Arora et al., 2013 [21], Goenharto et al., 2012 [22], Becker et al., 2004 [23]

	Dasgupta et al., 2021 [16]	Erwansyah et al., 2020 [17]	Alqahtani et al., 2019 [18]	Soto et al., 2017 [19]	Dash et al., 2016 [20]	Arora et al., 2013 [21]	Goenharto et al., 2012 [22]	Becker et al., 2004 [23]
Is the review question clearly and explicitly stated?	No	No	No	No	No	No	No	No
Were the inclusion criteria appropriate for the review question?	No	No	No	No	No	No	No	No
Was the search strategy appropriate?	No	No	Yes	Yes	No	No	No	No
Were the sources and resources used to search for studies adequate?	Unclear	Unclear	Unclear	Yes	Unclear	Unclear	Unclear	Unclear
Were the criteria for appraising studies appropriate?	Unclear	Unclear	Unclear	Yes	Unclear	Unclear	Unclear	Unclear
Was critical appraisal conducted by two or more reviewers independently?	Unclear	Unclear	Unclear	Unclear	Unclear	Unclear	Unclear	Unclear
Were there methods to minimize errors in data extraction?	No	No	No	No	No	No	No	No
Were the methods used to combine studies appropriate?	No	No	No	No	No	No	No	No
Was the likelihood of publication bias assessed?	No	No	No	No	No	No	No	No
Were recommendations for policy and/or practice supported by the reported data?	Yes	Yes	Yes	Yes	Yes	Yes	Yes	Yes
Were the specific directives for new research appropriate?	Yes	Yes	Yes	Yes	Yes	Yes	Yes	Yes
Total	2	2	3	5	2	2	2	2

Discussion

Four case reports and eight comprehensive literature reviews addressed the topic of the orthodontic treatment guidelines for individuals with mental illness by applying behavioral, pharmacological, or physical constraints. According to the findings of the present systematic review, only limited studies have examined the factors influencing how patients responded to orthodontic treatment and parental attitudes toward orthodontic care, which was consistent with the findings of a prior study [24]. There were no prospective controlled trials or systematic reviews of SHCN in any of those reviewed studies. The discrepancies in oral health between people with disabilities and the general population must be reduced, and eventually eliminated, due to the challenges that those with SHCN encounter when it pertains to maintaining basic oral hygiene measures and their likelihood of malocclusion. Parents are the single most significant motivator for orthodontic treatment and contribute an influential role in the acceptance of orthodontic care. Parents who want their children to receive orthodontic treatment primarily do so for optimized physical appearance and societal integration [25].

According to estimates, 12% to 18% of children worldwide have cognitive or motor challenges [19]. The term "special needs" is typically used to describe people who have disabilities related to development such as mental retardation, cerebral palsy, autism, ADHD, or Down syndrome, in addition to medically complex, at-risk individuals who are entitled to special care [21]. In the course of the pre-treatment assessment phase, professionals should properly record their medical history and comply with a healthcare provider. At the time of the initial session, information regarding their dental hygiene practices should be carefully gathered from the caretakers or parents [5]. A treatment strategy is to be developed, consisting of several different self-sufficient stages. An attempt has been made to segregate orthodontic mechanotherapy into modules to operate. A review should be conducted after each therapy module to determine whether the next step in the plan can be implemented. In a few instances, initial anxiety and apprehension gradually fade away, patient cooperation increases, and dental hygiene remains excellent. Then, the course of treatment may be continued in stages until the best possible outcome is obtained [23].

For patients with mental impairment, initial desensitization sessions were required before beginning orthodontic therapy. They are less able to comprehend complex instructions and newly acquired knowledge [26]. It is crucial to indicate that in most instances, customized adjustment of the appointments, application of behavior management approaches, such as tell-show-do, and positive and negative reinforcement suffice to accomplish the intended objectives of the various visits. It is now only a matter of determining which of the additional treatment options is the best for the least-tolerated patients [21]. In most other complex scenarios, patients with SHCN would typically require conscious sedation, a pharmacologically established state of relaxation while the patient is conscious. Due to their brief half-life and dementia-like effects, propofol and midazolam remain the most commonly utilized sedatives in the field of dentistry. Nonetheless, the most frequently reported side effect of these medications is somnolence. Further, as individuals with SHCN are prescribed several medications, the adverse effects of these drugs could also jeopardize their dental health [25].

The most complicated orthodontic procedures were performed under GA. The treatment must be carried out in a specialized operating theatre, which entails all the associated prospective intraoperative or postoperative pulmonary and cardiovascular problems. The likelihood of morbidity and the total cost is increased by these additional factors. The authors suggested the use of intravenous deep sedation as an alternative to GA, and they have shown that it is effective regardless of how challenging SHCN patients are. The relatively simple and painless procedures that are unique to orthodontics are considerably more conducive to it than GA. The anesthesiologist may administer it in the clinical setting as long as the dental office has been properly prepared for sedation [23]. GA is an exceptionally radical form of medical intervention, and it should never be used until other management techniques have been explored to offer dental care securely [14].

According to Taddei et al. [27] and Becker et al. [23], SHCN calls for additional appointments and treatment duration. They specifically contrasted SHCN with craniofacial deformities in individuals devoid of the condition and observed substantial variations in the frequency of sessions, length of therapy, age at commencement, and completion of the treatment. On the contrary, Blanck-Lubarsch et al. [28] who conducted a comparable study concluded that longer sessions are necessary rather than additional treatment time. The etiology of the malocclusion might not be completely resolved in all patients during the retention phase. As a result, post-treatment stability for skeletal vertical dysplasia or macroglossia may not be established. The retention period must be extended, and this must be made clear to the parent promptly. Attention has been brought to the effects of an unstable occlusion in children with Down syndrome owing to early aberrant involuntary contractions of the facial musculature or muscles of mastication, either by myofunctional or orthodontic interventions [19,23]. Moreover, the meticulous use of orthodontic appliances helps to prevent self-inflicted harm [14]. Considering the difficulty in communication experienced by individuals with mental disabilities, the dentist should pay close attention to hand gestures and verbal directives. Communication should be repeated slowly in simple language [16].

In the course of their orthodontic care, these patients should visit a hygienist frequently. During orthodontic therapy, it may be imperative to perform gingivectomy in individuals with gingival hyperplasia. The surgical procedure in individuals with SHCN will be challenging, and the parents should be made aware of this in advance. A favorable occlusion could be achieved with the minimum amount of treatment time with fixed appliances [29]. The most aggravating factor in this aspect may be the paucity of training for those professionals and dental teams. The critical elements for successful orthodontic treatment were empowering parents, understanding the constraints of these patients, and establishing an appealing clinical setting [25].

Cutting-edge innovations provide novel opportunities for SHCN individuals seeking treatment for malocclusion. These include less invasive surgical techniques, removable clear aligners, implants, self-ligating brackets, and superior dental hygiene practices. Temporary anchorage appliances that assist in moving specific teeth are also included. The enhancement of the self-esteem associated with an appealing smile has the potential to benefit individuals with SHCN as comparable to the general population [30].

Because there were scanty studies available to be included in the present systematic review, it was challenging to draw broad conclusions from the information gathered. The reviews that were chosen were purely narrative, and their results were based on the limited number of studies that included subjective observations and without any statistical comparisons.

## Conclusions

With numerous complementary therapeutic techniques available, the treatment of a mentally challenged patient necessitates specific attention. Parental cooperation and patient motivation are crucial components of a successful treatment regimen. A better prognosis is determined by the choice of appropriate orthodontic mechanotherapy along with the utilization of an array of behavior modification modalities and the availability of a team with expertise. In order to enhance the quality of our postgraduate services in hospitals, it is also important to include in orthodontists' residency training and curriculum the aims of behavioral and pharmacological management as well as the significance of a multidisciplinary treatment strategy.
